# Biocompatibility of Portland Cement Modified with Titanium Oxide and Calcium Chloride in a Rat Model

**DOI:** 10.7508/iej.2016.02.010

**Published:** 2016-03-20

**Authors:** Narjes Hoshyari, Hossein Labbaf, Nooshin Jalayer Naderi, Ali Kazemi, Farshid Bastami, Maryam Koopaei

**Affiliations:** a * Endodontic Department, Dental School, Mazandaran University of Medical Sciences, Sari, Iran; *; b * Endodontic Department, Dental School, Shahed University, Tehran, Iran; *; c * Oral Pathology Department, Dental School, Shahed University, Tehran, Iran; *; d * Research Institute of Dental Sciences, Dental School, Shahid Beheshti University of Medical Sciences, Tehran, Iran; *; e * Oral Medicine Department, Dental School, Tehran University of Medical Sciences, Tehran, Iran *

**Keywords:** Biocompatibility, Mineral Trioxide Aggregate, Portland Cement

## Abstract

**Introduction:**

The aim of the present study was to evaluate the biocompatibility of two modified formulations of Portland cement (PC) mixed with either titanium oxide or both titanium oxide and calcium chloride.

**Methods and Materials:**

Polyethylene tubes were filled with modified PCs or Angelus MTA as the control; the tubes were then implanted in 28 Wistar rats subcutaneously. One tube was left empty as a negative control in each rat. Histologic samples were taken after 7, 15, 30 and 60 days. Sections were assessed histologically for inflammatory responses and presence of fibrous capsule and granulation tissue formation. Data were analyzed using the Fisher’s exact and Kruskal-Wallis tests.

**Result:**

PC mixed with titanium oxide showed the highest mean scores of inflammation compared with others. There was no statistically significant difference in the mean inflammatory grades between all groups in each of the understudy time intervals.

**Conclusion:**

The results showed favorable biocompatibility of these modified PC mixed with calcium chloride and titanium oxide.

## Introduction

The aim of endodontic treatment is to prevent or treat the apical periodontitis. Root end filling materials are in direct contact with periodontal tissues. Therefore, biocompatibility and nontoxicity are among their essential properties [1, 2].

Mineral trioxide aggregate (MTA) is developed by Professor Torabinejad *et al.* [3] in order to seal communication between the tooth and its surrounding tissues. It is used for repair of root perforations, root end filling, barrier formation in teeth with necrotic pulps and open apices, pulpotomy, pulp capping and root canal filling [4]. The major component of MTA is calcium ion, and it is derived primarily from tricalcium silicate, tricalcium aluminate, tri-calcium oxide, silicate oxide and bismuth oxide [5].

Despite numerous advantages of MTA, its major problems include long setting time, difficult handling, difficulty in removal after setting, presence of some toxic elements in the material composition and high cost [6, 7]. Therefore, new attempts are focused on introducing new substitute and testing new formulations [8]. According to a review, Portland cement (PC) has properties similar to MTA [9]. Also, it is more available and has lower cost compared to MTA; as a result it may be considered as an alternative for MTA in endodontic therapy [10, 11].

However, PC has some clinical shortcomings like having a long setting time and low radiopacity. It has been reported that addition of CaCl_2_ reduces the setting time and improves the physicochemical properties of PC [12]. According to the literature, bismuth oxide has been added to PC in order to enhance its radiopacity; but it diminished compressive strength of PC. Titanium oxide is a biocompatible radiopaque material, which has antimicrobial effects [13-15]. Titanium oxide can also improve the mechanical properties of the root canal sealers [16]. Therefore, the idea of adding titanium oxide to PC to improve the physical properties of PC can be the subject of a study. Since endodontic cements are used in direct contact with periodontal tissues, establishment of their biocompatibility is essential before clinical uses. 

The aim of this animal study was to compare the biocompatibility of MTA and modified PC (mixed with either calcium chloride or titanium oxide or both) in subcutaneous connective tissues of rat model.

**Figure1:**
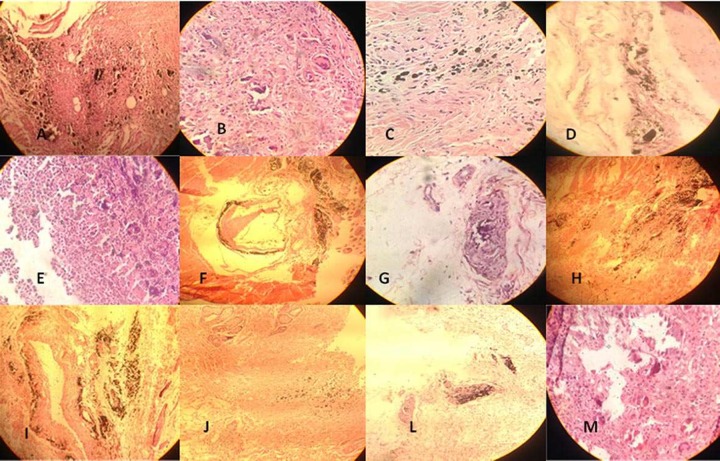
Rat subcutaneous tissue reaction the experimental tubes (H&E staining under 400× magnification). *A)* MTA group in 7^th^ day; *B)* MTA group in 15^th^ day; *C)* MTA group in 30^th^ day and *D)* MTA group in 60^th^ day; *E)* PCTC group in 7^th^ day; *F)* PCTC group in 15^th^ day; *G)* PCTC group in 30^th^ day and *H)* PCTC group in 60^th^ day; *I)* PCT group in 7^th^ day; *J)* PCT group in 15^th^ day; *L)* PCT group in 30^th^ day and *M)* PCT group in 60^th^ day

## Materials and Methods

The protocol of this experimental study was approved by Research Committee of School of Dentistry, Shahed University, and followed the policies and principles established by the Animal Welfare Act and the NIH Guide for Care and Use of Laboratory Animals. Twenty eight healthy 4 to 6-month male Wistar albino rats weighting 250 to 300 gr were divided into 4 groups (*n*=7). Rats were anesthetized with an intramuscular injection of ketamine (60 mg/kg) and xylazine (10 mg/kg). The dorsal skin was shaved and disinfected with 5% iodine solution. Four 15-mm-long incisions were made through the skin in a head to tail orientation using a #15 scalpel blade and pockets were prepared by undermining the incisions longitudinally by blunt dissection. 

The test groups were Angelus MTA (Angelus, Londrina, Paraná, Brazil) mixed according to the manufacturer, PC combined with titanium oxide (1.5 wt %) (PCT group) and PC combined with titanium oxide (1.5 wt %) and calcium chloride (10 wt %) (PCTC). Each cement was prepared by mixing 3 mg powder with 1 mL solution, and the paste was immediately placed in a sterile polyethylene tube (with 1.0 mm internal and 1.6 mm external diameters). Empty tubes were considered as the negative control group. Then, all understudy groups were randomly implanted in subcutaneous pocket of each rat. To prevent interactions of materials, the tubes were replanted at least 2 cm far from each other (two tubes in one side of the animals’ back and two in the other). Wounds were sutured. The animals were euthanized with an over dose of anesthetic solution 7, 15, 30, 60 days postoperatively, and the samples were harvested and fixed in 10% buffered formalin at a pH of 7.0 for 24-28 h before histological processing.

The tubes were bisected transversely and both halves were subsequently cut longitudinally with a sharp blade to allow the surfaces to maintain in contact with the processing solutions. The specimens were embedded in paraffin, sectioned serially to 3 µm slices and then stained with Hematoxylin and Eosin. Sections were evaluated under a light microscope (Carl Zeiss, Oberkochen, Germany), under 400× and 100× magnifications by an experienced blind pathologist.

Reactions in the tissues that were in contact with the material at the opening of the tube were scored as follows [17]: *score 1*, no or few inflammatory cells (no reaction) in microscopic field; *score 2*, less than 25 inflammatory cells (mild reaction) in microscopic field; *score 3*, 25 to 125 inflammatory cells (moderate reaction) in microscopic field; and *score 4,* 125 or more inflammatory cells (severe reaction) in microscopic field.

Fibrous capsules were classified as “thin” or “thick” if the thickness was <150 µm or >150 µm, respectively [17]. Observing the presence of calcification and granulation tissue formation were reported. Differences between the four sets of data were statistically analyzed using the Kruskal-Wallis and Fisher’s exact tests. The level of significance was set at 0.05.

## Results

Histological findings are presented in Figure 1. Empty tubes caused few or no inflammatory reactions in subcutaneous connective tissues. After 7 days, MTA group displayed mild inflammatory response which reduced to no-to-mild reaction after 15 and 30 days. These findings subsided by the 60^th^ day to no reaction (Figure 1A to 1D). 

**Figure2:**
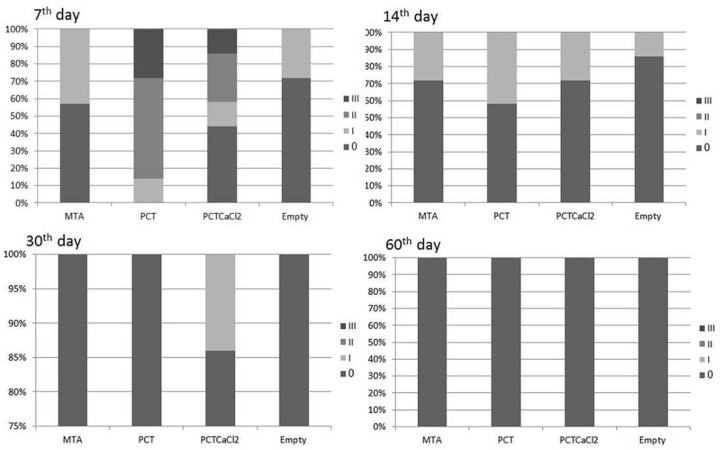
Distribution of inflammation grades (IG) in percentage after 7, 15, 30 and 60 days postoperatively (*PC: Portland cement, PCT: PC+titanium oxide, PCTC: PC+TiO*_2_*+Calcium chloride*

PCTC group reported mild to moderate response after 7 days, which reduced to no-to-mild reaction after 15 and 30 days, and no reaction at 60 days, postoperatively (Figure 1E to 1H). Also, PCT group revealed moderate to severe inflammatory reactions at 7 days, postoperatively. This response was reduced to no-to-mild at 15 and 30 days and no reaction at 60 days, postoperatively (Figure 1 and 2).

There was a statistically significant difference in the inflammation score among groups after 7 days (*P*<0.05). After 7 days, PCT group showed the highest mean scores of inflammation and MTA group showed the least (Figure 1I to 1M). After 15, 30 and 60 days, there was no statistically significant difference between mean inflammation scores among all groups (*P*>0.05).

According to histological assessments, it was demonstrated that areas of dystrophic calcification observed in study groups were similar in different times and no statistically significant difference was observed (*P*>0.05).

Formation of granulation tissue in PCT and PCTC groups was more than MTA group after 7 days (*P*>0.05), but it was similar after 15, 30 and 60 days (*P*<0.05). 

Incidence of fibrous capsule after 7 days was more in two modified PC groups compared to MTA and it was statistically significant between MTA and PCT groups (*P*<0.05). In other understudy times, it was similar between all groups (Figure 2).

## Discussion

Several studies have confirmed the similarities between MTA and PC, considering their physical, biological or microbiological properties [18, 19]. MTA and PC have similarities according to the x-ray diffraction analysis. After using both of them as direct pulp capping materials in rat teeth, apposition of reparative dentin occurred in both groups [20]. Esterla *et al.* [21] reported that PC had pH and antimicrobial activity similar to MTA. 

Chemical constitution and compressive strength of PC in comparison with MTA has been tested in the literature. It was demonstrated that PC has favorable physical and chemical properties, and its chemical composition was close to MTA [21, 22]. Addition of CaCl_2_ is planned to reduce the setting time and improve the physicochemical properties of PC [12]. Addition of a radiopaque agent to the components of cement is the main modification in formulation of MTA [23]. Alternative radiopacifiying materials comprise barium sulfate, titanium oxide/zinc oxide, gold powder and silver alloy [24]. Moreover, although addition of bismuth oxide gradually increases the relative porosity of the set material from 15 to 31%, it has been demonstrated that the more bismuth oxide was added into an endodontic PC-based material from 0 to 10 wt %, the less mechanical strength was obtained from 82 to 40 MPa [25]. 

Recently, titanium oxide has been introduced as a new material in dentistry because of its catalytic activity and biocompatibility [16, 26-28]. It has been reported that root canal sealers containing titanium oxide has antimicrobial components [13, 15]. Also, titanium oxide restorative materials improved the antibacterial and mechanical properties such as fracture toughness, compressive strength, flexural strength and micro-tensile bond strength [14].

Biocompatibility is described as the ability of a material to achieve an appropriate host response regarding a specific purpose [29]. It is rationale that testing the biologic potential of new dental materials is an essential issue before using it in contact with body [30]. The current study evaluated the biocompatibility of two modified PCs implanted into subcutaneous rat tissue. Since rat subcutaneous implantation studies are acceptable models for this assessment [31], this model was selected. Polyethylene tubes were used to implant the materials; in fact, in comparison with direct application of the material, using tubes helps to reach stability in material-tissue interface [32]. In this study, empty tubes caused few or no inflammatory reactions in subcutaneous connective tissues similar to previous studies [32].

According to the results of the current study, both modified PCs demonstrated favorable tissue reactions defined by absence of severe inflammatory reactions and the presence of a fibrous capsule [17]. Adding calcium chloride and titanium oxide to PCs showed inflammatory response after 7 days, but biocompatibility of these two modified PCs were favorable and similar with MTA in other understudy times. As an illustration, severity of inflammatory response was more in PCT compared to PCTC, but it decreased in both groups to the same level during different time intervals; in other words, addition of calcium chloride increased the biocompatibility by accelerating the setting time, and it seems that severe inflammatory response to PCT after 7 days may be the result of its prolonged setting time. 

Regarding the formation of fibrous capsules, MTA group revealed more fibrous capsules than two modified PCs and it was significant between MTA and PCT. This finding is in agreement with previous studies [17]. The formation of fibrous capsules indicates that these materials are tolerated by tissues [32]. Yaltirik *et al.* [32] showed formation of a thin fibrous capsule around implanted polyethylene tubes filled with MTA 30 days after implantation. 

Our results demonstrated that Angelus MTA has favorable biocompatibility which is in consistent with the literature [3, 33, 34]. For instance, Holland *et al.* [3] reported similar histologic results from the connective tissue reaction of subcutaneously implanted dentin tubes filled with MTA, PC and calcium hydroxide in rat. Furthermore, when MTA was used as a direct pulp cap or pulpotomy material, induction of hard tissue formation in pulpal tissues has been observed [35]. However, the addition of additives such as titanium oxide and CaCl_2_ are not involved in hard tissue deposition mechanism of MTA and PC [3, 32]. In fact, dystrophic calcification in MTA group has been demonstrated 7 days after implantation [32]. 

## Conclusion

In conclusion, our results showed that PCs modified with titanium oxide and calcium chloride have appropriate biocompatibility and may be suitable substitutes for MTA. We suggest further studies on the effects of modified PCs as alternatives to MTA to reach evidence-based conclusions.
